# Association between age at initial diagnosis and post-metastasis mortality among women with recurrent metastatic breast cancer in China

**DOI:** 10.1186/s12885-022-09454-y

**Published:** 2022-04-09

**Authors:** Yuxin Xie, Qiheng Gou, Yingjie Zhang, Keqi Xie, Dan Zheng, Chuanxu Luo, Jiaojiao Suo, Xiaorong Zhong, Ting Luo

**Affiliations:** 1 Department of Medical Oncology of Cancer Center, West China Hospital, Sichuan University, Chengdu, China; 2 Laboratory of Molecular Diagnosis of Cancer, Clinical Research Center for Breast, West China Hospital, Sichuan University, Chengdu, Sichuan China; 3 Department of Radiation Therapy, West China Hospital, Sichuan University, Chengdu, China; 4 Departments of Anesthesiology, Mianyang Central Hospital, Mianyang, Sichuan China; 5 Department of Medical Oncology of Cancer Center, Clinical Research Center for Breast, West China Hospital, Sichuan University, 37 Guoxue Xiang, Wuhou District, Chengdu, 610041 China

**Keywords:** Age, Recurrent metastatic breast cancer, Prognosis, Survival, Cohort study

## Abstract

**Background:**

Little is known about whether age at initial diagnosis influences the prognosis of recurrent metastatic breast cancer (rMBC). Here, we analyzed the association between age at initial diagnosis and rMBC mortality in China.

**Methods:**

A total of 1636 women diagnosed with rMBC between 1989 and 2020 at West China Hospital, Sichuan University were included in this study. The age at initial diagnosis was categorized as young (≤ 40 years), middle-aged (41–64 years) and elderly (≥ 65 years). Post-metastasis mortality was the primary outcome and its associated factors were analyzed by Cox proportional hazards models.

**Results:**

During a median follow-up of 5.2 years after initial diagnosis of breast cancer, 620 deaths were identified. Compared with middle-aged patients, elderly patients had a 70% increased risk of post-metastasis mortality (95%CI, 1.24–2.33) after adjusting for demographics, tumor characteristics and treatment modes. Similarly, elderly patients were associated with a 75% increased risk of post-metastasis mortality (95%CI, 1.19–2.59) compared with young patients. Subgroup analyses also showed similar trends.

**Conclusion:**

Our findings suggest that in breast cancer, elderly patients at initial diagnosis face a higher risk of post-metastasis mortality.

**Supplementary Information:**

The online version contains supplementary material available at 10.1186/s12885-022-09454-y.

## Background

Recently, the International Agency for Research on Cancer releases the latest estimates on the global burden of cancer and provides estimates of incidence and mortality for 2020. One of the most shocking changes is the rapid growth in the newly diagnosed cases of breast cancer, which has replaced lung cancer as the world's largest cancer. According to the latest estimated data in 2020, there will be 2.26 million new breast cancers and 670,000 cancer deaths around the world [1].

Globally, due to the aging of the population, the World Report on Ageing and Health estimates that the number of people older than 60 years will double by 2050. More than 50% of breast cancers are diagnosed in patients older than 60 years in the USA. This proportion is still nearly 30% in China [2, 3]. The global cancer burden will increase by 50% by 2040 compared with 2020 [4]. Thus, cancer has become a major public health issue worldwide, especially for elderly patients.

The rate of metastasis for breast cancer is still increasing. Approximately 20%-30% of patients with early breast cancer will experience distant metastatic relapse and 90% of cancer-related deaths are attributed to metastasis [5, 6]. Distant metastasis can lead to a dramatic reduction of 5-year overall survival rate to only approximately 25%, compared with 80% for breast cancer patients without metastasis [6, 7]. Current treatments for metastatic breast cancer include palliation with chemotherapeutic, hormonal, and biologic agents, neither of which has an adequate effect on improving survival [8].

Age is one of the major risk factors for breast cancer. The World Health Organization and Medicare define the elderly as individuals older than 65 years [9]. Some studies have shown a striking relationship between increasing age at initial diagnosis and an increased risk of disease-specific mortality in breast cancer [10, 11]. However, an opposite association was observed in other studies [12, 13]. Although the overall prognosis of breast cancer has been substantiated in numerous studies, few studies focused on survival in patients with recurrent metastatic breast cancer (rMBC). Utilizing a large-scale cohort of patients in China diagnosed during 1989–2020, we investigated the association of age at initial diagnosis with the risk of post-metastasis mortality (PMM).

## Methods

### Study population

A retrospective cohort study was conducted using the Breast Cancer Information Management System (BCIMS). The BCIMS database covers virtually all patients diagnosed with invasive breast cancer at West China Hospital, Sichuan University from 1989 to 2020 and collects information on demographics, clinical characteristics, laboratory examinations, treatments, and follow-up visits [14].

A cohort of 15,660 breast cancer cases diagnosed between 1989 and 2020 was reviewed in this study. Of these, only 2,059 cases of metastatic breast cancer were included. Then 10 male patients, 18 patients with primary bilateral breast cancer and 395 patients with de novo metastatic disease were excluded, leaving 1,636 female patients with rMBC in the final analysis. Recurrent metastatic breast cancer was defined as distant recurrence or metastatic and identified by annual follow-up in patients with primary stage I-III breast cancer, while patients with locoregional recurrence were excluded from this analysis.

This study was approved by the Clinical Trial and Biomedical Ethics Committee at West China Hospital, Sichuan University (reference number: 2012–130). Informed consent forms were obtained from all patients.

### Construction of variables

The primary independent variable of interest in this study was age at initial diagnosis of breast cancer, stratified as young (≤ 40 years old), middle-aged (41–64 years) and elderly (≥ 65 years), after reference to previous studies [15–17].

The demographics included calendar year at diagnosis, ethnic group, insurance type and educational level (as proxies for socioeconomic status), marital status, and body mass index (BMI). Insurance type was classified as urban (i.e. Urban Resident-Based Basic Medical Insurance Scheme [URBMI], Urban Employee-Based Basic Medical Insurance Scheme [UEBMI], and/or commercial insurances) and rural (i.e. New Rural Cooperative Medical Scheme) schemes [14]. According to the recommendation to Asian populations [18], BMI was classified into < 23 kg/m^2^ (non-overweight) and ≥ 23 kg/m^2^ (overweight). Clinical characteristics included menopausal status, comorbidity, histological type, histological grade, tumor stage, hormone receptor status including both estrogen receptors (ER) and progesterone receptors (PR), human epidermal growth factor receptor 2 (HER2) status, Ki-67 level, molecular subtype and metastatic sites. Molecular subtypes of breast cancer are divided into four categories by joint HR/HER2/Ki-67 status: Luminal A (HR + /HER2-/Ki-67 < 14%), Luminal B (HR + /HER2 + or ER + /HER2-/Ki-67 ≥ 14%), HER2-positive (HR-/HER2 +), and triple-negative (HR-/HER2-) [19]. Metastatic sites were classified as bone, brain, visceral (lung, liver, and intracavity lymph nodes, etc.), contralateral breast, and others (skin, soft tissue, distant lymph nodes, etc.). Local recurrence was defined as the recurrence in any part of the ipsilateral breast, chest wall, and regional lymph nodes. Treatment modes were categorized as surgery, any chemotherapy, any radiotherapy, any hormonal therapy, and any targeted therapy.

### Post-metastasis mortality, overall mortality and breast cancer-specific survival

All patients were actively followed up through telephone and medical visits until death or June 22^st^, 2020, whichever came first. The underlying cause of death was ascertained from the medical records, whenever possible, or informed by the immediate family members.

The post-metastasis (PMS) or overall survival (OS) was defined as the time elapsed between the first onset of metastasis or primary breast cancer diagnosis and the date of death due to any cause, respectively. And the breast cancer-specific survival (BCSS) was calculated as the time from the date of diagnosis to the date of death attributed to breast cancer. All patients still alive were censored at the date of last follow-up. Metastatic-free interval was defined as time between date of diagnosis of primary breast cancer and date of diagnosis of first distant recurrence metastatic.

### Statistical analysis

The demographics, clinical characteristics and treatment modes of patients in different age groups were described. Pearson's chi-square test was used to compare the differences in proportions among the age groups.

The differences in survival of patients among age groups were assessed. Survival curves were obtained using the Kaplan–Meier method and the curves were compared with log rank test.

The cumulative rates and 95% confidence intervals (CIs) of post-metastasis (PMM), overall (OM) and breast cancer-specific (BCSM) mortality by age was measured and plotted using a competing risk model. Hazard ratios (HRs) and 95% CIs were then estimated from Cox regression by contrasting age groups. In Cox regression analysis, the demographics (calendar year at diagnosis, ethnic group, insurance type and educational level and marital status), clinical characteristics (comorbidity, histological type, tumor stage, hormone receptor status, HER2 status, Ki-67 level and recurrent/metastatic sites), and treatment modes (surgery, chemotherapy, radiotherapy, hormonal therapy and targeted therapy) were adjusted.

All analyses were performed in STATA statistical software (version 14; STATA, College Station, TX). *P* value < 0.05 indicated statistical significance.

## Results

### Patients’ characteristics

Overall, 1636 patients (median age, 47 years; range, 22–89 years) were included in the study, of which 363 were younger than 40 years at diagnosis (22.19%; median age, 35 years), 1,173 were aged 40 to 64 years (71.70%; median age, 49 years), and 100 were aged 65 years or older (6.11%; median age, 69 years). A significant age-associated increase was observed in patients with urban schemes, married and postmenopausal status, higher BMI and comorbidities at diagnosis as well as less well-educated level and lower proliferative disease (Ki-67 < 14%). In addition, tumors were more likely to be HER2-negative in young and elderly patients. The administration of chemotherapy and radiotherapy significantly decreased with age, whereas the proportion of hormonal therapy increased significantly in young and elderly patients (both *P*< 0.05; Table 1). Further, we analyzed the palliative treatments which the women received after metastasis. The results showed that elderly patients generally use less systematic treatments, compared to young and middle-aged ones (Table 2).

**Table 1:** Characteristics of patients with recurrent metastatic breast cancer by age (years) at diagnosis from a breast cancer cohort in China between 1989 and 2020

Characteristics	All (*n*= 1,636)	< 40 (*n*= 363)	40–64 (*n*= 1,173)	≥ 65 (*n*= 100)	*P*
Year at diagnosis					0.099
1989–2007	490 (29.95)	98 (27)	354 (30.18)	38 (38.00)	
2008–2020	1,146 (70.05)	265 (73)	819 (69.82)	62 (62.00)	
Ethnic group					0.799
Minority	28 (1.71)	6 (1.65)	20 (1.71)	2 (2.00)	
Han	1,472 (89.98)	322 (88.71)	1,058 (90.2)	92 (92.00)	
Unknown	136 (8.31)	35 (9.64)	95 (8.1)	6 (6.00)	
Insurance type					0.004
Urban schemes	1,159 (70.84)	249 (68.6)	823 (70.16)	87 (87.00)	
Rural schemes	462 (28.24)	112 (30.85)	337 (28.73)	13 (13.00)	
Unknown	15 (0.92)	2 (0.55)	13 (1.11)	0	
Education (years)					< 0.001
≤ 6	241 (14.73)	23 (6.34)	200 (17.05)	18 (18.00)	
7–9	485 (29.65)	106 (29.2)	364 (31.03)	15 (15.00)	
10–12	293 (17.91)	66 (18.18)	205 (17.48)	22 (22.00)	
> 12	250 (15.28)	95 (26.17)	141 (12.02)	14 (14.00)	
Unknown	367 (22.43)	73 (20.11)	263 (22.42)	31 (31.00)	
Marital status					< 0.001
Non-married	23 (1.41)	15 (4.13)	8 (0.68)	0	
Married	1,487 (90.89)	315 (86.78)	1,078 (91.9)	94 (94.00)	
Unknown	126 (7.7)	33 (9.09)	87 (7.42)	6 (6.00)	
BMI (kg/m^2^)					< 0.001
< 23	616 (37.65)	175 (48.21)	409 (34.87)	32 (32.00)	
≥ 23	654 (39.98)	117 (32.23)	500 (42.63)	37 (37.00)	
Unknown	366 (22.37)	71 (19.56)	264 (22.51)	31 (31.00)	
Menopausal status					< 0.001
Premenopausal	918 (56.11)	345 (95.04)	572 (48.76)	1 (1.00)	
Postmenopausal	713 (43.58)	16 (4.41)	599 (51.07)	98 (98.00)	
Unknown	5 (0.31)	2 (0.55)	2 (0.17)	1 (1.00)	
Comorbidity					0.016
No	1,435 (87.71)	332 (91.46)	1,021 (87.04)	82 (82.00)	
Yes	201 (12.29)	31 (8.54)	152 (12.96)	18 (18.00)	
Hormone receptor status					0.232
Negative	525 (32.09)	103 (28.37)	391 (33.33)	31 (31.00)	
Positive	1,015 (62.04)	242 (66.67)	708 (60.36)	65 (65.00)	
Unknown	96 (5.87)	18 (4.96)	74 (6.31)	4 (4.00)	
HER2 status					< 0.001
Negative	949 (58.01)	243 (66.94)	640 (54.56)	66 (66.00)	
Positive	398 (24.33)	67 (18.46)	312 (26.6)	19 (19.00)	
Unknown	289 (17.67)	53 (14.6)	221 (18.84)	15 (15.00)	
Ki-67 level					< 0.001
< 14%	222 (13.57)	37 (10.19)	155 (13.21)	30 (30.00)	
≥ 14%	1,129 (69.01)	262 (72.18)	812 (69.22)	55 (55.00)	
Unknown	285 (17.42)	64 (17.63)	206 (17.56)	15 (15.00)	
Molecular subtype					0.001
Luminal A	160 (9.78)	41 (11.29)	99 (8.44)	20 (20.00)	
Luminal B	714 (43.64)	173 (47.66)	502 (42.8)	39 (39.00)	
HER2-positive	187 (11.43)	28 (7.71)	150 (12.79)	9 (9.00)	
Triple-negative	289 (17.67)	65 (17.91)	205 (17.48)	19 (19.00)	
Unknown	286 (17.48)	56 (15.43)	217 (18.5)	13 (13.00)	
Tumor size^a^					0.078
T1	295 (18.03)	62 (17.08)	206 (17.56)	27 (27.00)	
T2	759 (46.39)	161 (44.35)	549 (46.8)	49 (49.00)	
T3	161 (9.84)	43 (11.85)	113 (9.63)	5 (5.00)	
T4	199 (12.16)	40 (11.02)	147 (12.53)	12 (12.00)	
Unknown	222 (13.57)	57 (15.7)	158 (13.47)	7 (7.00)	
Nodal status^a^					0.734
N0	417 (25.49)	98 (27)	287 (24.47)	32 (32.00)	
N1	439 (26.83)	102 (28.1)	313 (26.68)	24 (24.00)	
N2	273 (16.69)	59 (16.25)	199 (16.97)	15 (15.00)	
N3	386 (23.59)	77 (21.21)	285 (24.3)	24 (24.00)	
Unknown	121 (7.4)	27 (7.44)	89 (7.59)	5 (5.00)	
Metastatic sites					
Bone					0.117
No	1,101 (67.30)	244 (67.22)	799 (68.12)	58 (58.00)	
Yes	535 (32.70)	119 (32.78)	374 (31.88)	42 (42.00)	
Brain					0.124
No	1,431 (87.47)	317 (87.33)	1,020 (86.96)	94 (94.00)	
Yes	205 (12.53)	46 (12.67)	153 (13.04)	6 (6.00)	
Viscera					0.060
No	701 (42.85)	140 (38.57)	510 (43.48)	51 (51.00)	
Yes	935 (57.15)	223 (61.43)	663 (56.52)	49 (49.00)	
Contralateral breast					0.065
No	1,503 (91.87)	323 (88.98)	1,086 (92.58)	94 (94.00)	
Yes	133 (8.13)	40 (11.02)	87 (7.42)	6 (6.00)	
Others					0.089
No	1,318 (80.56)	291 (80.17)	938 (79.97)	89 (89.00)	
Yes	318 (19.44)	72 (19.83)	235 (20.03)	11 (11.00)	
Local recurrence					0.622
No	1,178 (72.00)	258 (71.07)	844 (71.95)	76 (76.00)	
Yes	458 (28.00)	105 (28.93)	329 (28.05)	24 (24.00)	
Histological type					0.071
Ductal	1,368 (83.62)	304 (83.75)	975 (83.12)	89 (89.00)	
Others	132 (8.07)	34 (9.37)	89 (7.59)	9 (9.00)	
Unknown	136 (8.31)	25 (6.89)	109 (9.29)	2 (2.00)	
Histological grade					0.265
I/II	318 (19.44)	65 (17.91)	226 (19.27)	27 (27.00)	
III	685 (41.87)	161 (44.35)	484 (41.26)	40 (40.00)	
Unknown	633 (38.69)	137 (37.74)	463 (39.47)	33 (33.00)	
Surgery					0.767
No	63 (3.85)	16 (4.41)	44 (3.75)	3 (3.00)	
Yes	1,573 (96.15)	347 (95.59)	1,129 (96.25)	97 (97.00)	
Anyradiotherapy					0.002
No	807 (49.33)	158 (43.53)	586 (49.96)	63 (63.00)	
Yes	829 (50.67)	205 (56.47)	587 (50.04)	37 (37.00)	
Any chemotherapy					0.006
No	242 (14.79)	41 (11.29)	177 (15.09)	24 (24.00)	
Yes	1,394 (85.21)	322 (88.71)	996 (84.91)	76 (76.00)	
Any hormonal therapy					0.012
No	605 (36.98)	114 (31.4)	460 (39.22)	31 (31.00)	
Yes	1,031 (63.02)	249 (68.6)	713 (60.78)	69 (69.00)	
Any targeted therapy					0.096
No	1,431 (87.47)	309 (85.12)	1,029 (87.72)	93 (93.00)	
Yes	205 (12.53)	54 (14.88)	144 (12.28)	7 (7.00)	

BMI was classified into < 23 kg/m^2^ (non-overweight) and ≥ 23 kg/m^2^ (overweight) according to the recommendation to Asian populations

^a^Initial primary breast tumor

*HER2* human epidermal growth factor receptor 2, *BMI* Body mass index

**Table 2:** Associations of age (years) at diagnosis with palliative therapy in patients with recurrent metastatic breast cancer from a breast cancer cohort in China between 1989 and 2020

All		< 40	40–64	≥ 65	*P*
**Regimens**					
	**No. ( %)**	**No. (%)**	**No. ( %)**	**No. ( %)**	
**Palliative chemotherapy**					< 0.001
** No/ unknown**	746 (45.60)	148 (40.77)	533 (45.44)	65 (65.00)	
** Yes**	890 (54.40)	215 (59.23)	640 (54.56)	35 (35.00)	
**Palliative radiotherapy**					0.259
** No/ unknown**	1,437 (87.84)	311 (85.67)	1,035 (88.24)	91 (91.00)	
** Yes**	199 (12.16)	215 (59.23)	138 (11.76)	9 (9.00)	
**Palliative hormonal therapy**					0.013
** No/ unknown**	1,055 (64.49)	212 (58.40)	772 (65.81)	71 (71.00)	
** Yes**	581 (35.51)	151 (41.60)	401 (34.19)	29 (29.00)	
**Palliative target therapy**					0.077
** No/ unknown**	1,494 (91.32)	326 (89.81)	1,071 (91.30)	97 (97.00)	
** Yes**	142 (8.68)	37 (10.19)	102 (8.70)	3 (3.00)	
**No. of regimens**					0.003
** One**	577 (35.27)	122 (33.61)	418 (35.64)	37 (37.00)	
** Tow**	375 (22.92)	102 (28.1)	261 (22.25)	12 (12.00)	
** Three**	147 (8.99)	39 (10.74)	103 (8.78)	5 (5.00)	
** Four**	11 (0.67)	3 (0.83)	8 (0.68)	0 (0.00)	
** Unknown**	526 (32.15)	97 (26.72)	383 (32.65)	46 (46.00)	
**One regimen**					0.125
** Only chemotherapy**	374 (64.82)	76 (62.29)	278 (66.51)	20 (54.05)	
** Only radiotherapy**	13 (2.25)	2 (1.64)	8 (1.91)	3 (8.11)	
** Only hormonal therapy**	183 (31.72)	41 (33.61)	128 (30.62)	14 (37.84)	
** Only target therapy**	7 (1.21)	3 (2.46)	4 (0.96)	0 (0.00)	
**Two regimens**					0.190
** Chemo- and radiotherapy**	60 (16.00)	17 (16.67)	42 (16.09)	1 (8.33)	
** Chemo- and hormonal**	239 (63.73)	65 (63.73)	166 (63.60)	8 (66.67)	
** Chemo- and target**	59 (15.73)	15 (14.71)	43 (16.48)	1 (8.33)	
** Radiotherapy and hormonal**	10 (2.67)	2 (1.96)	6 (2.30)	2 (16.67)	
** Radiotherapy and target**	0 (0.00)	0 (0.00)	0 (0.00)	0 (0.00)	
** Hormonal and target**	7 (1.87)	3 (2.94)	4 (1.53)	0 (0.00)	

### Age at initial diagnosis and survival of breast cancer

**Fig. 1:**
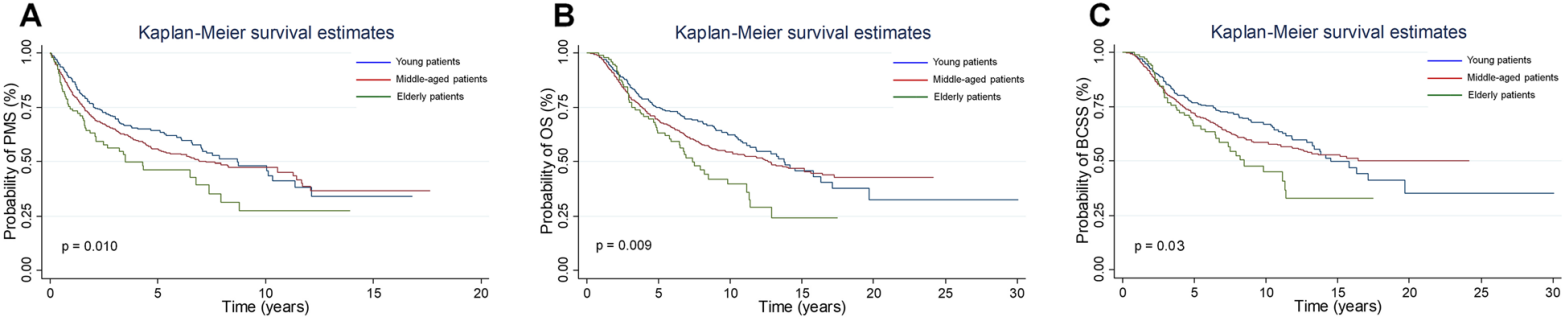
Kaplan–Meier estimates of post-metastasis survival (**A**), overall survival (**B**) or breast cancer-specific survival (**C**) in different age groups

During follow-up after breast cancer diagnosis (median 5.2 years, interquartile range, 2.8–8.8 years), 620 rMBC patients (37.89%) died and 548 of them were attribute to breast cancer. The median follow-up after metastatic recurrence was 2.4 years (interquartile range, 1.0–3.9 years). Median PMS for young patients was 8.69 years, while for middle-aged and elderly patients was 7.24 and 3.49 years, respectively. Ten-year PMS rates were 46.0%, 47.6% and 27.5% in groups of young, middle-aged and elderly age, respectively. Results showed that elderly patients had a worse PMS than young or middle-aged ones. Similar patterns were noticed for overall and breast cancer-specific survival (Fig. 1).

### Age at initial diagnosis and mortality of breast cancer

**Fig. 2:**
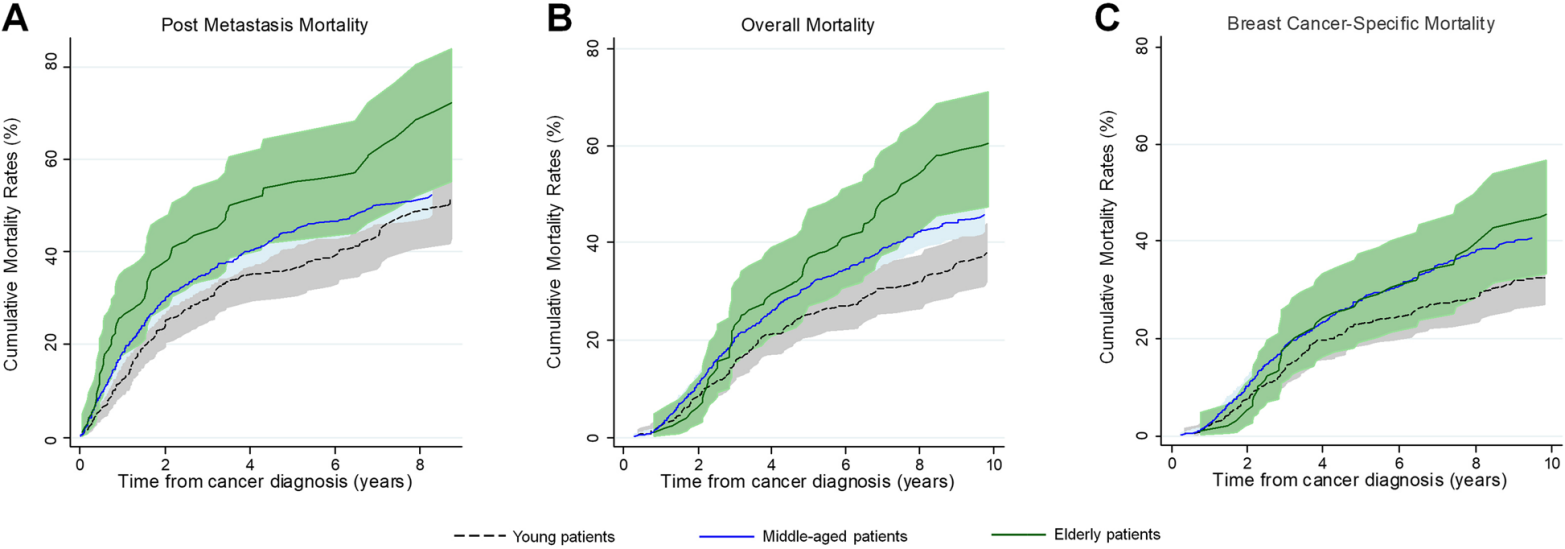
Cumulative mortality rates of post-metastasis mortality (**A**), overall mortality (**B**) or breast cancer-specific mortality (**C**) in different age groups

**Fig. 3:**
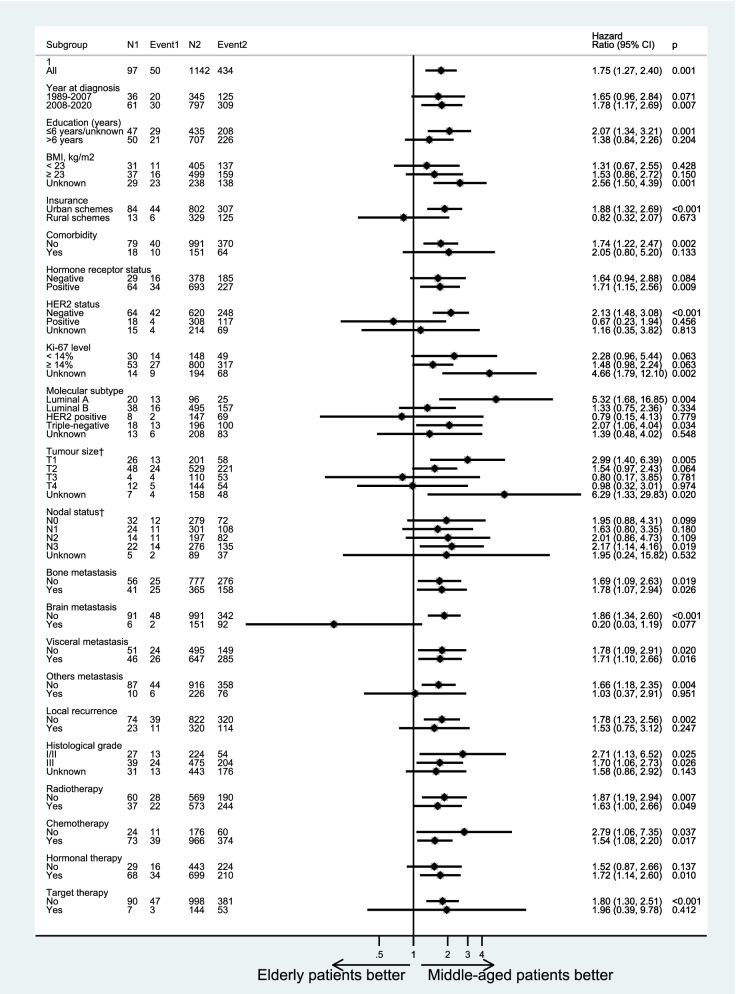
Forest plots for risks of post-metastasis mortality compared between elderly and middle-aged patients, by stratification factors

**Fig. 4:**
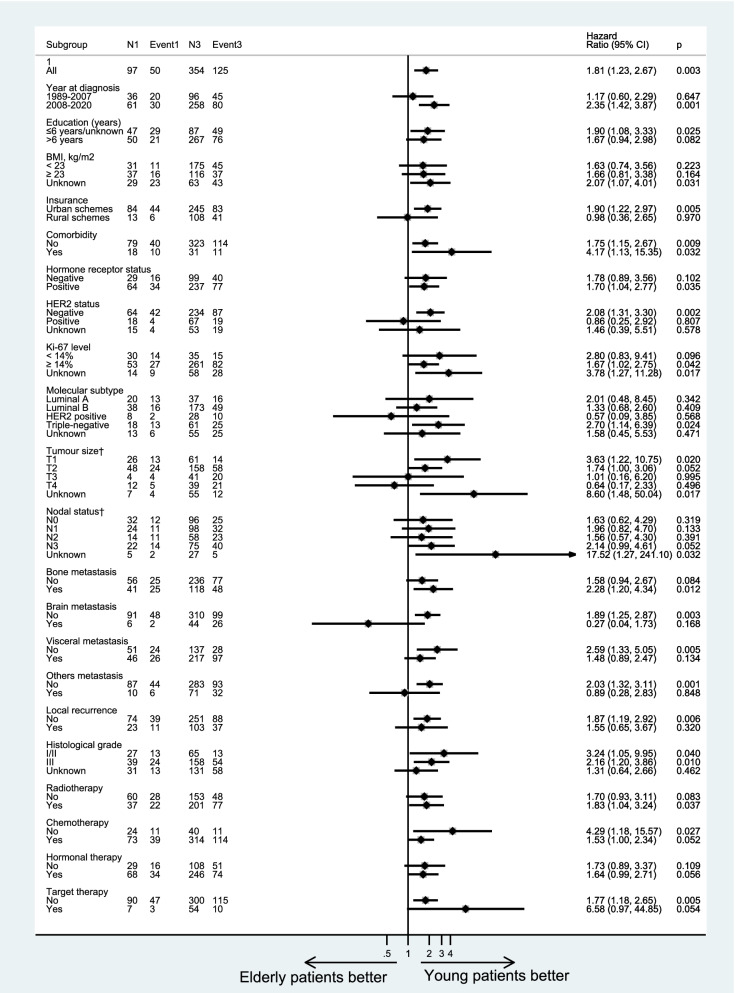
Forest plots for risks of post-metastasis mortality compared between elderly and young patients, by stratification factors

The cumulative rates of PMM were higher among elderly patients, compared with young and middle-aged patients (Fig. 2). Similar patterns were noticed for overall mortality. When adjusting for demographics, clinical characteristics and treatment modes, elderly patients had a 70% increased risk of PMM (95%CI: 14% to 87%) compared with middle-aged patients (Fig. 3). Similarly, when compared with young patients, patients with elderly age had a 75% increased risk of PMM (95%CI: 11% to 82%) (Fig. 4). Similar trends were found for OM (Supplementary Figures S1 and S2) and BCSM (Supplementary Figures S3 and S4). Additionally, considering age is a continuous variable, we analyzed and confirmed the significantly prognostic role of age as a continuous variable in breast cancer (Supplementary Table 1).

### Age at initial diagnosis and mortality of breast cancer by subtype stratification

In subtype analysis, with control for demographics, clinical characteristics and treatment modes, the association between age and prognosis was much more significant (Figs. 3 and 4). After stratification by molecular subtypes, elderly patients had a higher death risk in rMBC with luminal A subtype (HR = 5.32; 95%CI: 1.68–16.85) and triple-negative subtype (HR = 2.07; 95%CI: 1.06–4.04), compared with middle-aged patients. However, elderly patients showed a higher death risk (HR = 2.70; 95%CI: 1.14–6.39) only in triple-negative subtype, compared with young patients. It was noteworthy that, after stratification by brain metastatic site, elderly patients presented with a better prognosis than young and middle-aged patients, although there was no statistical difference due to the small sample size.

For treatment mode subgroups, administration of hormonal therapy for patients, elderly patients had a 72% increased risk of mortality (95%CI: 1.14–2.60) than middle-aged ones. In radiotherapy subgroup, elderly patients showed a higher death risk (HR = 1.83; 95%CI: 1.04–3.24) compared with young ones.

In addition, patients in subgroup like diagnosis year in 2008–2020, less well-education, urban schemes, breast cancer with hormone receptor-positive, HER2-negative, stage T1 or N3, the increased risk for the elderly patients still be significant. Similar patterns were found for OM (Supplementary Figures S1 and S2).

However, there was no subgroup difference between young and middle-aged patients in PMM, OM and BCSM (Supplementary Figures S5, S6 and S7, respectively).

## Discussion

To the best of our knowledge, this is the first study to demonstrate that elderly patients with recurrent and distant metastatic breast cancer are at increased risk of PMM, compared with the young and middle-aged groups in China. Importantly, this association is partly but not entirely explained by known prognostic indicators, including demographic factors, tumor characteristics, and cancer treatment. In addition, the correlation was not significant between the young and middle-aged groups.

The relationship between age and prognosis of breast cancer has always been controversial. Findings from several studies have shown that the percentage of deaths attributed to breast cancer decreased with increasing age [12, 13]. However, an opposite association was observed in other studies [10, 11]. Additionally, some results reveal that young and elderly breast cancer patients are more likely to have a poorer outcome than middle-aged patients [20, 21]. Here, our data further illustrated that the increasing age is associated with PMM and overall mortality in rMBC patients independent of clinical factors.

Several possible underlying mechanisms may help to explain the results presented in this study. First, elderly patients may experience poor socioeconomic status, including poor educational level and health insurance, and have limited access to medical service, which may lead to delayed diagnosis and suboptimal treatment [22]. This is also supported by our data that in poor educational level subgroup, elderly patients suffer an increased risk of PMM compared with young and middle-age patients. However, compared with rural health insurance, we only provide arguments that PMM increases with age in urban health insurance subgroup. In China, rural health insurance includes the New Rural Cooperative Medical Scheme (covering the residents of rural households and launched in 2003), while urban health insurance includes URBMI (covering the unemployed, children, and elderly and launched in 2007), and UEBMI (covering employees and launched in 1998) [14, 22]. Apparently, age is associated with the type of urban health insurance. Compared with UEBMI, URBMI is limited to a lower reimbursement cap and covers a narrower spectrum of diseases [23]. Thus, elderly patients with inadequate insurance face a greater financial burden and are less likely to afford out-of-pocket medical expenses for early diagnosis and advanced therapy, especially for rMBC patients who have already invested large funds to earlier stage disease [.24]. In line with that, our data highlights the urgent need of promoting social support to significantly improve breast cancer prognosis in elderly patients.

Next, for analysis of molecular typing subgroup, our results showed that elderly patients were associated with increased risk of PMM in triple-negative breast cancer (TNBC) subgroup, compared with either young or middle-aged patients. There may be multi-factorial explanations. A lot of age-related factors may play important roles in metastasis and prognosis, including accumulation of immune dysfunction, DNA damage, chronic inflammation, drug resistance, etc. [25]. Immune checkpoint blockade has been of greatest interest in TNBC due to its immunogenicity, as evidenced by the presence of tumor-infiltrating lymphocytes and elevated programmed cell death-ligand 1 expression relative to other subtypes. However, Sceneay et al. implicated that the tumor microenvironment in aged patients with TNBC showed decreased interferon cell signaling and antigen presentation, suggesting failed innate immune activation with age and age-related immune dysfunction as a mechanism of immunotherapy resistance [25]. Besides, chemotherapy is the main available systemic therapy for TNBC patients. Elderly patients with TNBC obtain similar benefits from adjuvant chemotherapy compared with young patients, but are at greater risk of toxicity. Furthermore, elderly patients may suffer a greater incidence of comorbidity, use of multiple medications, and preexisting dysfunction, such that treatment-related toxicity may dramatically affect their prognosis. These call attention to us that elderly patients with TNBC face a special tumor heterogeneity and major treatment challenge [26, 27].

The proportion of hormone receptor-positive breast cancers increases with age and is considered as the more favorable tumor biology, since hormone receptor-positive tumors tend to grow more slowly and frequently respond to hormonal therapy. Significant treatment benefits with endocrine therapy have been shown in the elderly patient population [28]. However, for the administration of hormonal therapy subgroup, our results showed that elderly patients had a 72% increased rate of PMM risk (95%CI: 1.14–2.60) than middle-aged ones, after exhaustive adjustment for clinical factors. We found that nearly 30% elderly patients with postmenopausal status still chose a selective estrogen receptor modulator, tamoxifen and toremifene. In addition, the hormonal therapy time was less than five years in 60% patients. A previous study has demonstrated that aromatase inhibitors (AIs) have been shown to be more effective than tamoxifen in the metastatic setting [29]. The American Society of Clinical Oncology clinical practice guideline recommends that women with node-positive breast cancer receive extended therapy, including an AI, for up to a total of 10 years of endocrine treatment [30]. A growing body of evidence has shown significantly prolonged progression-free survival and a manageable toxicity profile for first-line CDK4/6 inhibitor plus AI in patients with hormone receptor-positive/HER2-negative advanced breast cancer [31, 32]. Even for elderly patients, CDK4/6 inhibitor plus endocrine therapy is an effective, well-tolerated treatment [33]. Obviously, due to the long treatment period and other reasons, endocrine therapy seems to be difficult to maximize its effect for elderly patients. These may cause cancer-related mortality increase with age.

The International Society of Geriatric Oncology has assembled a task force to make evidence-based recommendations for the treatment of breast cancer patients in the elderly [34]. Furthermore, new combination regimens that target multiple pathways have shown efficiency in elderly patients who were previously resistant to endocrine therapies. Future studies may need to focus on the combinations to improve outcomes of elderly patients with rMBC. With the rapid development of endocrine therapy, we believe that better results will be obtained in the future.

A major strength of our study is the large-scale cohort design with virtually complete follow-up, largely limiting the common sources of bias. The rich information on demographics and clinical characteristics helped to disentangle the direct influence of age on PMM, from the influence through demographics, tumor characteristics and treatment modes. Then we can further analyze the detailed correlation among subgroups. Our study also has several limitations. First, this cohort is based on a single regional medical center, the findings may not be generalized to the entire population. Furthermore, inadequate data of treatment limited analysis of its effect on the correlation between age and prognosis.

## Conclusions

In conclusion, our findings suggest that elderly patients face a higher risk of PMM in China, which may provide the knowledge of prognostic and predictive markers that will allow individualized therapy for rMBC.

## Supplementary Information

**Additional file 1.**

## Data Availability

All data generated or analyzed during this study are included in this published article and its supplementary information files. The datasets generated and analyzed during the current study are available in the Breast Cancer Information Management System (BCIMS) database.

## Abbreviations

AI: Aromatase inhibitor
BCIMS: Breast Cancer Information Management System
BMI: Body mass index
BCSM: Breast cancer-specific mortality
BCSS: Breast cancer-specific survival
CI: Confidence interval
HER2: Human epidermal growth factor receptor 2
HR: Hazard ratio
OM: Overall mortality
OS: Overall survival
PMM: Post-metastasis mortality
PMS: Post-metastasis survival
rMBC: Recurrent metastatic breast cancer
TNBC: Triple-negative breast cancer
UEBMI: Urban Employee-Based Basic Medical Insurance Scheme
URBMI: Urban Resident-Based Basic Medical Insurance Scheme
