# Supplementary material for: Association between age at initial diagnosis and post-metastasis mortality among women with recurrent metastatic breast cancer in China
Source: BMC Cancer. 2022 Apr 9;22:385. doi: 10.1186/s12885-022-09454-y (PMC8994897; doi:10.1186/s12885-022-09454-y)
Supplement: Supplementary file 1 — Additional file 1. [file 12885_2022_9454_MOESM1_ESM.doc]

**Supporting Information**

**Association between age at initial diagnosis and post-metastasis mortality among women with recurrent metastatic breast cancer in China**

Yuxin Xie^1,2,#^, Qiheng Gou^1,#^, Yingjie Zhang^3^, Keqi Xie^4^, Dan Zheng^1,2^, Chuanxu Luo^1,2^, Jiaojiao Suo^1,2^, Xiaorong Zhong^1,2^,Ting Luo^1,2,∗^

^1^Department of Medical Oncology of Cancer Center, West China Hospital, Sichuan University, Chengdu, China;

^2^Laboratory of Molecular Diagnosis of Cancer, Clinical Research Center for Breast, West China Hospital, Sichuan University, Chengdu, Sichuan, China;

^3^Department of radiation therapy, West China Hospital, Sichuan University, Chengdu, China;

^4^Departments of Anesthesiology, Mianyang Central Hospital, Mianyang, Sichuan, China.

^#^Equal contribution to the work.

^∗^Correspondence to: Dr. Ting Luo, Department of Medical Oncology of Cancer Center, Clinical Research Center for Breast, West China Hospital, Sichuan University, 37 Guoxue Xiang, Wuhou District, Chengdu 610041, China.

Tel: +86-28-85422685; Email: [tina621@163.com](mailto:tina621@163.com)

**Contents:**

Page 2-8 Supporting Information Figure S1 – 7

Page 9 Supporting Information Table S1

**Supplementary Figure S1.** Forest plots for risks of overall mortality compared between elderly and middle-aged patients, by stratification factors.


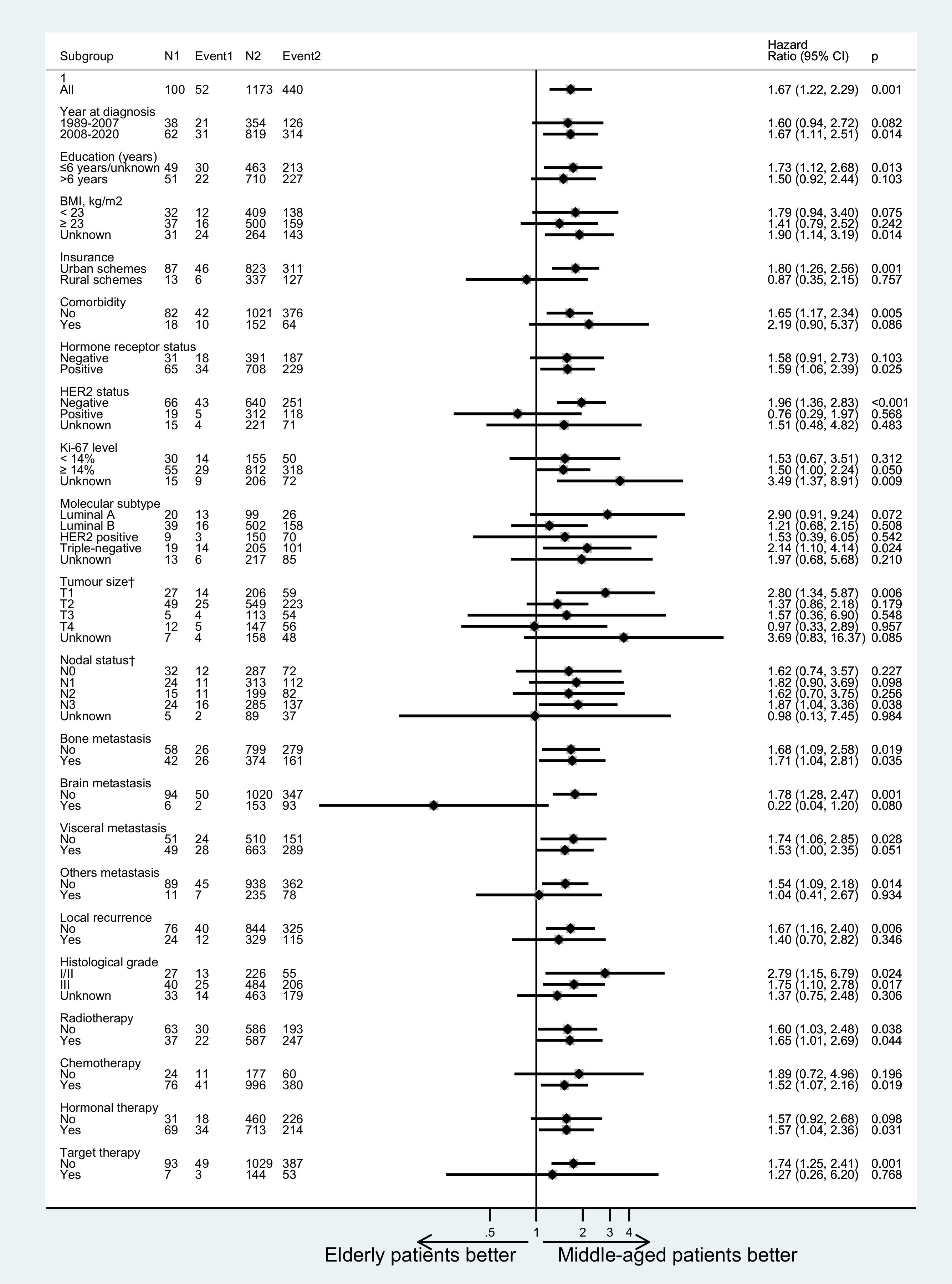


**Supplementary Figure S2.** Forest plots for risks of overall mortality compared between elderly and young patients, by stratification factors.


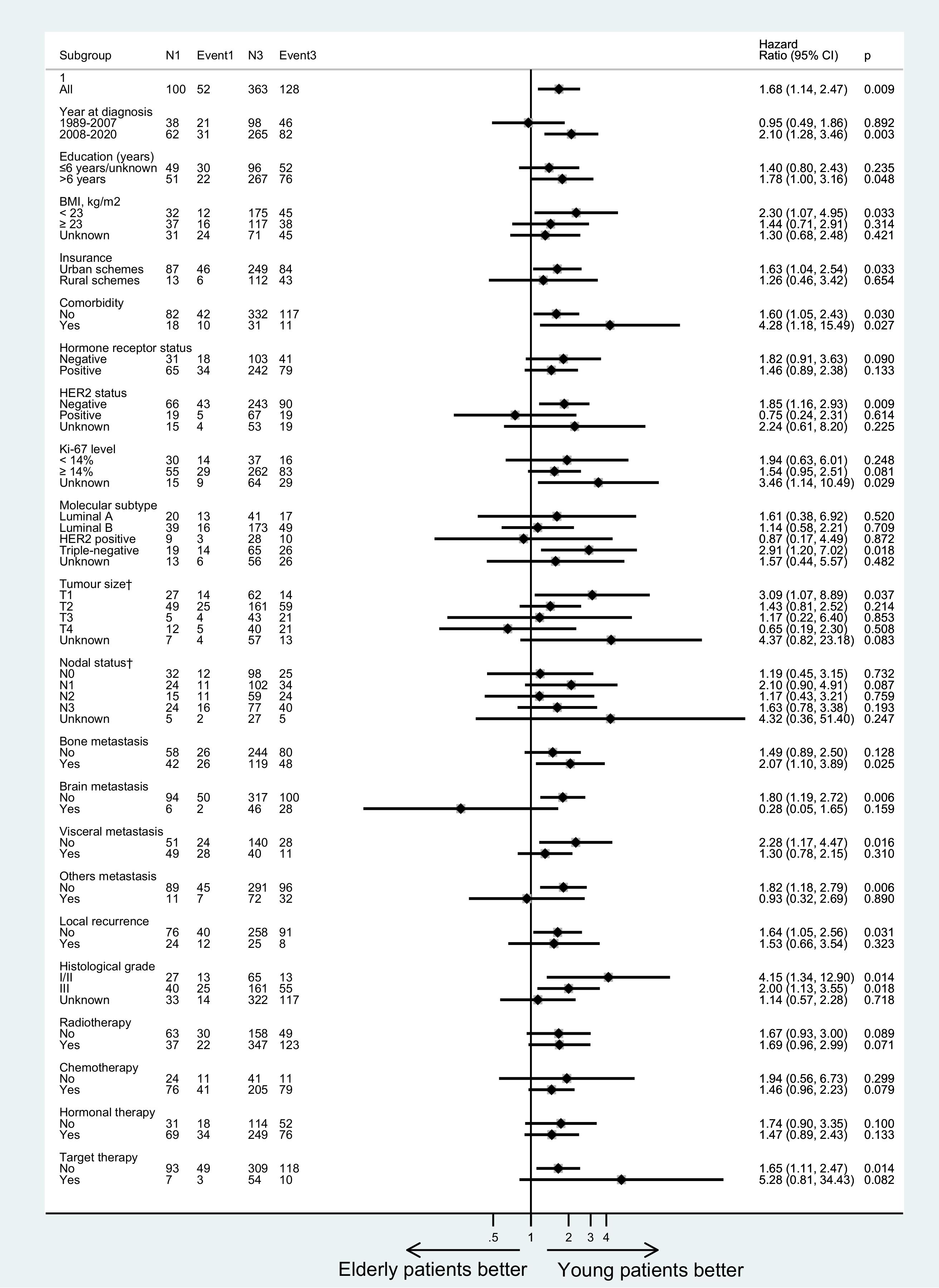


**Supplementary Figure S3.** Forest plots for risks of breast cancer-specific mortality compared between elderly and middle-aged patients, by stratification factors.


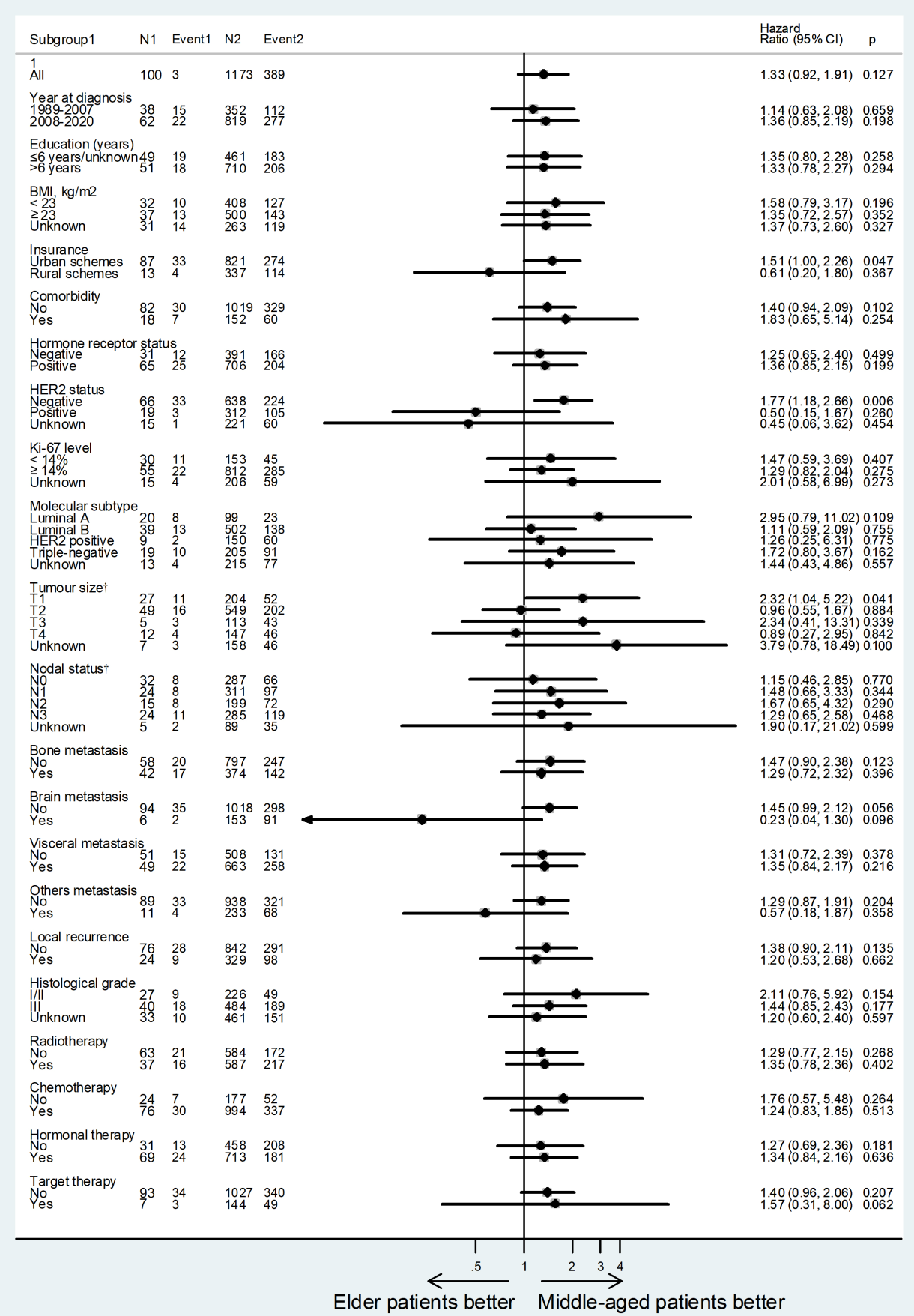


**Supplementary Figure S4.** Forest plots for risks of breast cancer-specific mortality compared between elderly and young patients, by stratification factors.


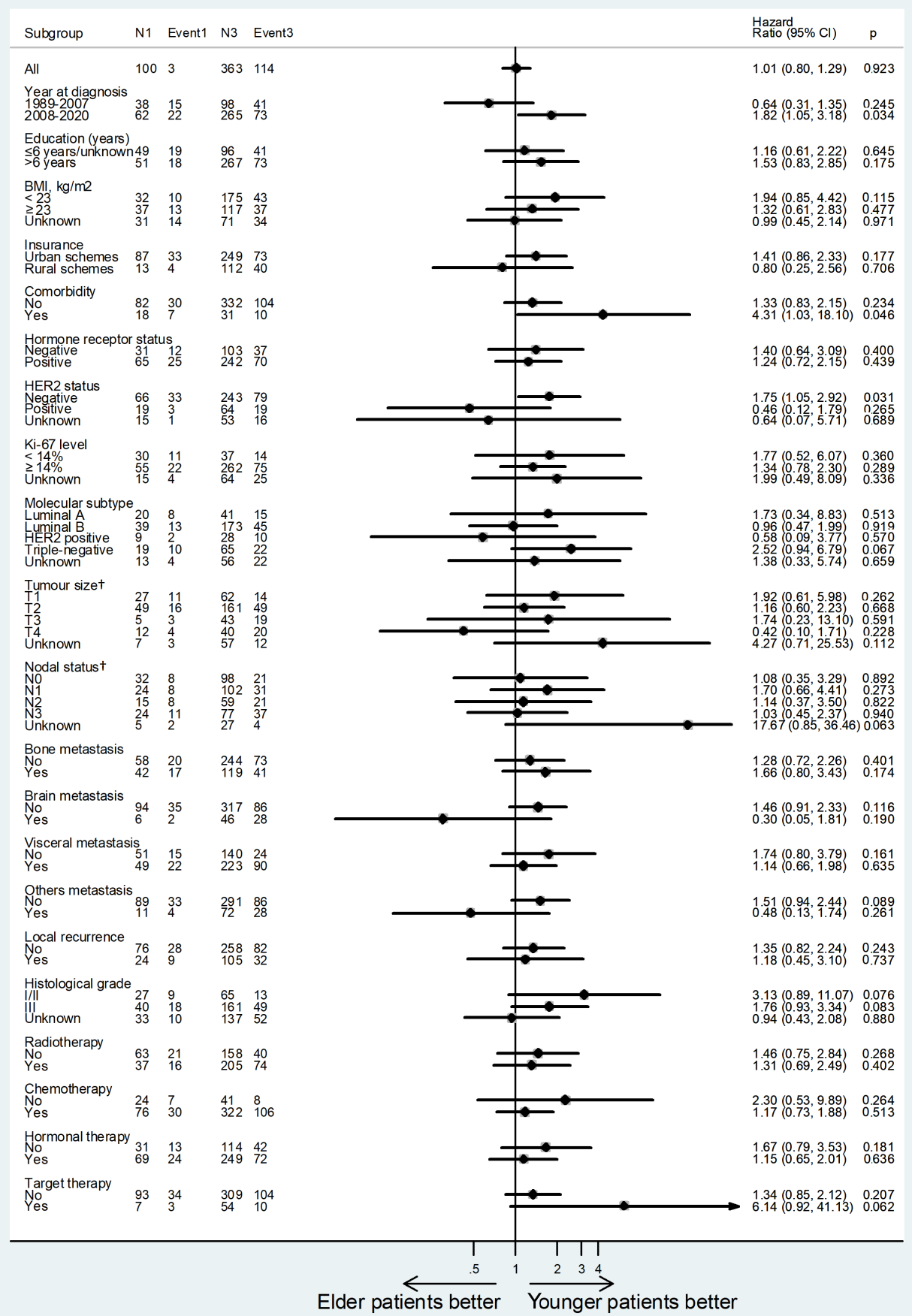


**Supplementary Figure S5.** Forest plots for risks of breast post-metastasis mortality compared between young and middle-aged patients, by stratification factors.


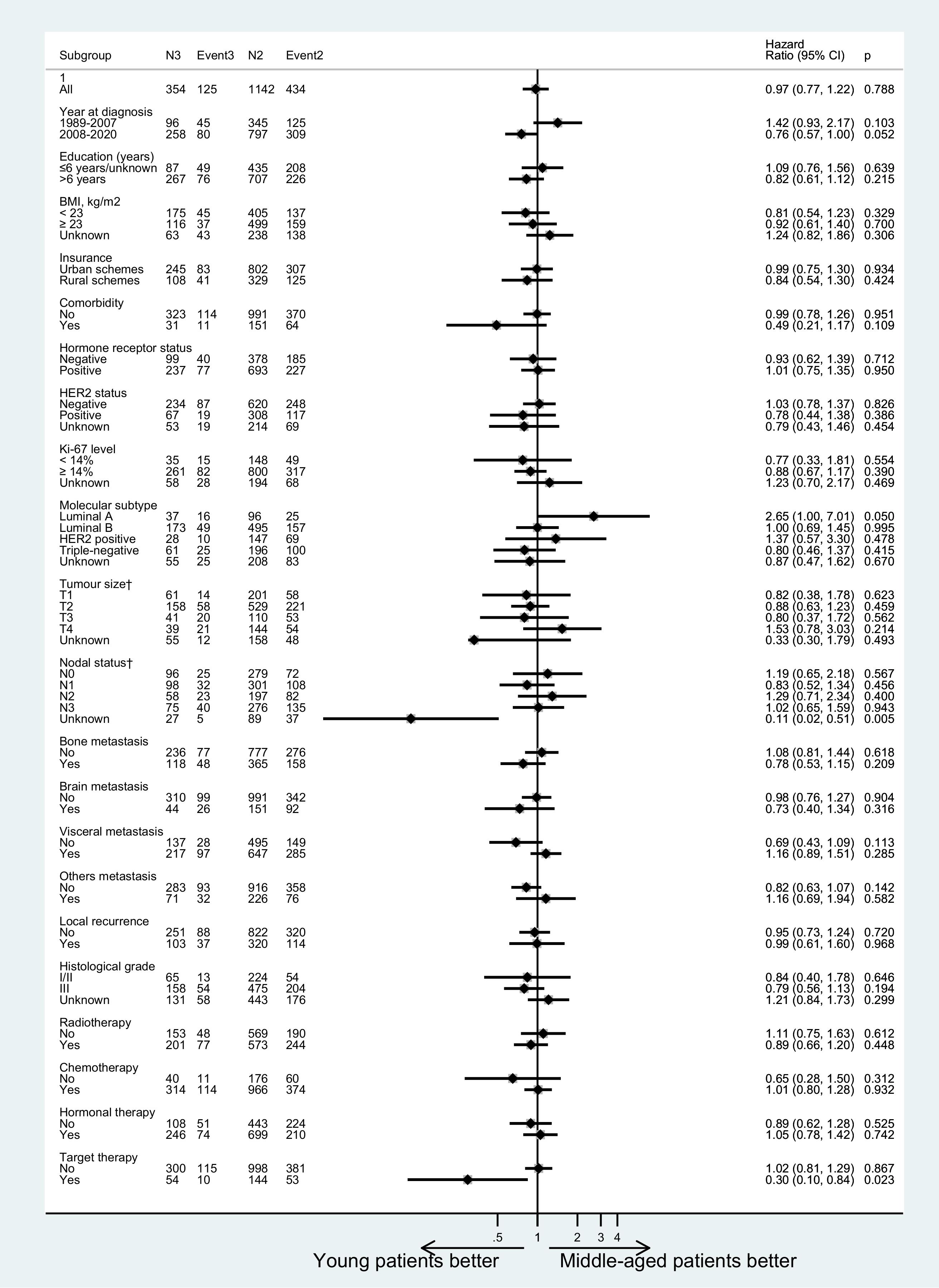


**Supplementary Figure S6** Forest plots for risks of overall mortality compared between young and middle-aged patients, by stratification factors.


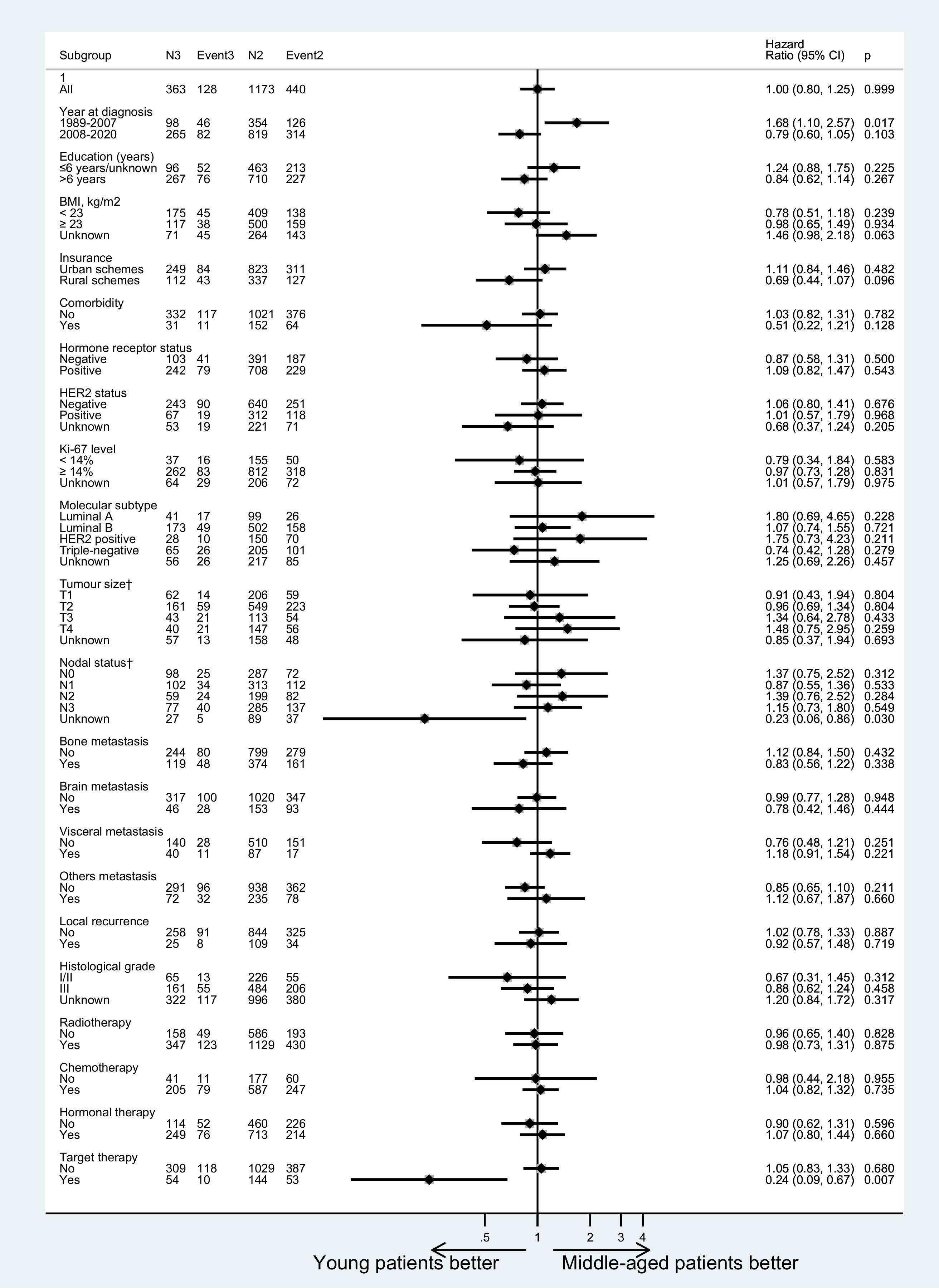


**Supplementary Figure S7.** Forest plots for risks of breast cancer-specific mortality compared between young and middle-aged patients, by stratification factors.


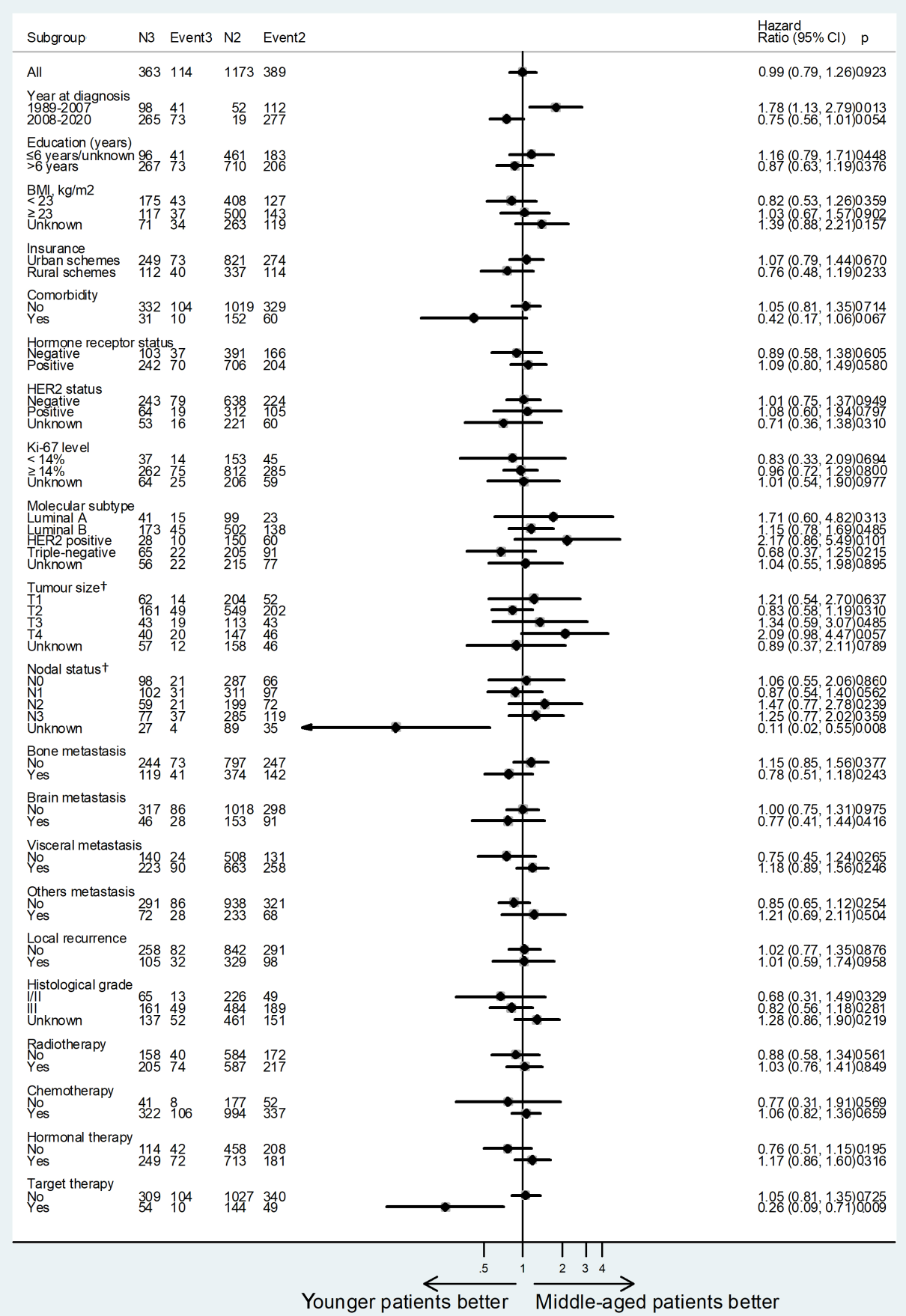


**Table: S1 Associations of age as a** continuous variable with risks of post-metastasis, overall or breast cancer-specific mortality.

|  | **Model^†^** | |
| --- | --- | --- |
|  | **HR (95% CI)** | **P** |
| **Post-metastasis mortality** |  |  |
| Age as continuous variable | 1.018 (1.006-1.030) | 0.002 |
| **Overall mortality** |  |  |
| Age as continuous variable | 1.016 (1.004-1.027) | 0.008 |
| **Breast cancer-specific mortality** |  |  |
| Age as continuous variable | 1.015 (1.002-1.027) | 0.018 |

NOTE. HR, hazard ratio; CI, confidence interval.

^†^HRs were adjusted for calendar year at diagnosis, ethnic group (majority or minority/unknown), education (>6 years, or ≤6 years/unknown), insurance type (urban schemes, rural schemes/unknown), marital status (married or non-married), comorbidity (no or yes), hormone receptor status (negative, positive, or unknown), HER2 status (negative, positive, or unknown), Ki-67 level (<14%, ≥14%, or unknown), histological type (ductal or other types/unknown), tumor stage (I, II, III, IV, or unknown), surgery (yes or no), chemotherapy (yes or no), radiotherapy (yes or no), hormonal therapy (yes or no), and targeted therapy (yes or no).
